# Pigtail catheter drainage and surgery in severe acute pancreatitis

**DOI:** 10.1002/jgh3.12182

**Published:** 2019-04-11

**Authors:** Manish Kumar, Sakthivel Chinnakkulam Kandhasamy, Ashok Kumar Sahoo, Anandhi Amaranathan, Mangala Goneppanavar, Vishnu Prasad Nelamangala Ramakrishnaiah

**Affiliations:** ^1^ Department of Surgery Jawaharlal Institute of Postgraduate Medical Education and Research (JIPMER) Puducherry India; ^2^ Department of Pathology Mahatma Gandhi Medical College and Research Institute Puducherry India

**Keywords:** open necrosectomy, pigtail drainage, severe acute pancreatitis, step‐up approach

## Abstract

**Background and Aim:**

Severe acute pancreatitis is initially managed with conservative treatment. Patients who failed conservative management were effectively treated with pigtail drainage. Factors predicting surgery remain uncertain.

**Methods:**

A total of 58 patients with severe acute pancreatitis presenting to JIPMER Hospital were studied and managed with a step‐up approach. In this prospective observational study, patients were divided into three groups based on the final mode of treatment received: conservative, pigtail alone, and surgery groups.

**Results:**

Of 58 patients, 30 patients were managed with conservative treatment, 20 patients with pigtail alone, and 8 patients underwent surgical treatment after pigtail failure. Overall sepsis reversal was achieved in 22 of 28 (78.5%) patients: 18 were in the pigtail alone group, and 4 were in surgery group, which was statistically significant (*P* = 0.03). Respiratory failure was the most common organ failure, 68.9%, and overall mortality was 8.62 in this study. On univariate analysis, absence of sepsis reversal within 2 weeks of pigtail insertion is a predictor of need of surgery. Other significant findings were higher catheter‐related complications in the surgery group (*P* = 0.01) and a 100% unimicrobial infection in the surgery group (*P* = 0.02). Overall mortality was 8.6%, which did not differ significantly between the groups.

**Conclusion:**

The step‐up approach avoided unnecessary intervention, and 52% patients recovered by conservative treatment alone. Sepsis reversal within 2 weeks of pigtail insertion can be used as a predictor of surgery in the early course of severe acute pancreatitis managed by the step‐up approach.

## Introduction

Acute pancreatitis is one of the most common gastrointestinal (GI) emergencies presenting to a tertiary care hospital.^1^ Gall stone disease and alcoholism are the leading causes and account for 80% of all cases.^2^ Of patients, 20% present with severe acute pancreatitis, with a mortality rate of up to 40%. This mortality can increases to 70% in cases of infected necrosis.^3^

Patients with acute necrotizing pancreatitis were traditionally managed by open necrosectomy. It is associated with a high rate of complications in 52–88% of patients and with mortality in 18–36%, with a risk of long‐term pancreatic insufficiency.^4^ Due to these reasons, less‐invasive alternatives to open necrosectomy are being explored, such as percutaneous catheter drainage, endoscopic transgastric procedures, and minimally invasive retroperitoneal approaches.

Recent studies have suggested that patients with severe acute pancreatitis with necrotic fluid collection can be managed with percutaneous drainage alone, and surgical intervention can be avoided in 30–100% of patients.^5^ The patients with acute necrotising pancreatitis (PANTER) trial showed the feasibility and success of the step‐up approach compared to open necrosectomy in a randomized controlled manner.^6^ In our institute, all patients with severe acute pancreatitis are managed by conservative treatment initially, followed by percutaneous intervention if needed. Open nectrosectomy is performed if the patient deteriorates. Hence, we tried to find the factors predicting the need for surgery, along with the efficacy of pigtail drainage, in patients presenting with severe acute pancreatitis managed using the step‐up approach.

## Methods

The study was carried out in the Department of Surgery, JIPMER, Puducherry from September 2014 to August 2016. The institute's Human Ethics Committee approval was obtained for the study, and informed consent was obtained from all patients. All provisions of the Declaration of Helsinki were followed in this study. All patients were managed using the step‐up approach with the intent to treat, and only observations were noted.

### *Inclusion and exclusion criteria*

Patients above 18 years of age having severe acute pancreatitis with one or more of the following: Bedside Index of Severity in Acute Pancreatitis (BISAP) score > 2, modified computed tomography (CT) severity index ≥ 8, Acute Physiology, Age, Chronic Health Evaluation (APACHE) II score ≥ 8, and/or persistent single or multiple organ failures were included. Patients with acute or chronic pancreatitis requiring surgery at presentation and those with a pigtail catheter before presentation were excluded.

### *Data collection*

Data were collected and entered in prespecified proforma at admission and serially after. Demographics (age, gender), etiology**,** complete blood count, urea and creatinine, ultrasonography, contrast‐enhanced computed tomography**,** clinical and severity scores**,** pigtail catheter details**,** number of total drains**,** total number of days, and drain fluid microbiology were noted. Blood and fungal culture data were not recorded for the purpose of this study. C‐reactive protein and procalcitonin were not available in our institute free of cost.

All patients included in the study were managed using the step‐up approach and were divided at the end of study into three groups: Group 1: patients managed conservatively, Group 2: patients managed with pigtail catheter drainage alone, and Group 3: patients who underwent surgery after pigtail drainage. Initially, all patients received medical management. Then, an image‐guided pigtail catheter (standard 16 Fr size) was used to drain the infected fluid in the second step as per indications. Drain tube block was managed with flushing, and slippage was managed with image‐guided repositioning. Patients not improving on percutaneous drainage were taken for surgery as the third step. In our institute, open necrosectomy was considered the third step as minimally invasive surgery is currently lacking in the emergency set‐up.

The timeframe for the conservative group was not defined. A decision was made to intervene depending on the clinical condition of the patient, such as those who did not improve on medical management with persisting fever, leukocytosis, worsening or new‐onset organ failure, presence of gas in pancreatic bed, or presence of symptomatic fluid collection. Patients who did not improve on medical management with persisting fever, leukocytosis, worsening or new‐onset organ failure, presence of gas in pancreatic bed, or presence of symptomatic fluid collection were put on pigtail catheter drainage. Patients with persistent/worsening sepsis after pigtail drainage in the form of persistently raised or increasing leukocyte count, persistent/worsening organ failure or new‐onset organ failure, ongoing sepsis, inadequate drainage of collection and necrosis, failure to thrive, and/or catheter‐related complications were considered for pancreatic necrosectomy.

Patients were observed primarily for sepsis reversal with pigtail catheter drainage, proportion of patients requiring surgical necrosectomy after initial pigtail drainage, and identification of factors that predicted the need for surgery in patients initially treated with pigtail drainage. Secondarily, groups were monitored for morbidity in terms of length of hospital stay, number of catheters required, number of interventions required, catheter‐related complications, and mortality.

Criteria for catheter removal included drain output of 10 mL/day of nonpurulent fluid for two consecutive days after adequate flushing and ensuring patency with normal amylase levels, no residual collection on serial imaging, and clinical recovery. Reversal of sepsis with a pigtail catheter was monitored using the following factors: clinical defervescence and reversal of leukocytosis and sepsis‐related organ failure with or without resolution of the necrotic cavity.

Organ failure was decided based on modified Marshall scoring (Table 1). Ongoing sepsis was defined as a patient having an intermittent low‐grade fever, with waxing and waning of leukocyte in the form of simmering infection and incomplete resolution of the necrotic cavity on imaging. Failure to thrive was defined as sepsis reversal with incomplete resolution of the necrotic cavity and associated with diminished appetite, inadequate oral intake, failure to gain weight, and being unable to carry out routine activities.

**Table 1:** Modified Marshall scoring system for organ dysfunction^7^

	Score				
Organ system	0	1	2	3	4
Renal: serum creatinine (mg/dL)	<1.4	1.4–1.8	1.9–3.6	3.6–4.9	>4.9
Cardiovascular	<90	<90	<90	<90, pH < 7.3	<90, pH < 7.2
Systolic BP (mmHg)		Fluid responsive	Not fluid responsive		
Respiratory (PaO_2_/FiO_2_)	>400	301–400	201–300	101–200	<101
For nonventilated patients, the FiO_2_ can be estimated as below					
Supplemental oxygen (L/min)	FiO_2_ (%)				
Room air	21				
2	25				
4	30				
6–8	40				
9–10	50				

A score of ≥2 in any system defines the presence of organ failure.

BP, blood pressure.

### *Statistical analysis*

The statistical analysis was conducted using SPSS version 24 for windows (IBM SPSS Statistics for Windows, Armonk, NY, IBM Corp., USA). Kolmogorov–Smirnov tests of normality were used to test the normality of data. Means for normally distributed data were compared using the Student t‐test for two groups. anova was used for more than two groups. For skewed data, the Mann–Whitney test was applied. Proportions were compared using the chi‐square test or Fischer's exact test wherever applicable. Univariate analysis was performed using logistic regression for predictors of surgery.

## Results

In this prospective study, of 58 patients, 30 patients were included in Group 1 and 20 patients in Group 2, and the remaining 8 patients were assigned to Group 3.

### *Age, gender, and etiology*

There was no significant difference between the ages of the groups. Gender distribution was highly skewed, with only two females and 56 males in the whole study who belonged to the conservative group. Alcohol abuse was the most common cause of pancreatitis in 81% of cases (47/58). Gallstone was the second most common cause, with six patients (10%) in total (Table 2). The difference in gender distribution could be explained by the socially acceptable fact that alcohol abuse is uncommon in females in India.

**Table 2:** Comparison among the groups

Characteristics	Group 1 (*n* = 30)	Group 2 (*n* = 20)	Group 3 (*n* = 8)	*P*‐value 1	*P*‐value 2
Age in years (mean ± SD)	38.7 ± 12	38.7 ± 12	36.1 ± 6	0.99	0.57
Etiology (%)					
Alcohol	23 (76.7)	17 (85)	7 (87.5)		
Gall stones	5 (16.7)	1 (5)	—		
Others	2 (6.7)	2 (10)	1 (12.5)		
Clinical and severity scores					
BISAP (mean ± SD)	2.13 ± 0.7	2.3 ± 0.6	2.25 ± 0.7	0.39	0.88
Modified CTSI (mean ± SD)	7.64 ± 1.54	7.88 ± 1.6	7.75 ± 1.67	0.68	0.85
APACHE II at admission	10.4 ± 4.1	9.05 ± 3.6	9.38 ± 1.3	0.22	0.26
APACHE II at first pigtail	—	9.7 ± 2.8	10.36 ± 2.97	—	0.68
APACHE II at surgery	—	—	7.5 ± 2.0	—	—
Length of hospital stay	12 ± 7.2	21.1 ± 11.7	70.1 ± 24.2	Both 0.0002	
Necrosis, *n* (%)					
No necrosis	7 (23.3)	6 (30)	2 (25)	0.74	1.0
Less than 30%	14 (46.7)	6 (30)	1 (12.5)	0.37	0.6
30–50%	4 (13.3)	2 (10)	3 (37.5)	1.0	0.12
More than 50%	5 (16.7)	5 (25)	2 (25)	0.49	1.0
Organ failure					
No organ failure, *n* (%)	9 (30)	5 (25)	1 (12.5)	0.75	0.64
Single organ failure, *n* (%)	13 (43.3)	12 (60)	5 (62.5)	0.38	1.0
Multiorgan failure, *n* (%)	8 (26.7)	3 (15)	2 (25)	0.48	0.60
Renal failure, *n* (%)	10 (33.3)	4 (20)	2 (25)	0.35	1.0
Respiratory failure, *n* (%)	19 (63.3)	14 (70)	7 (87.5)	0.76	0.63
CVS failure, *n* (%)	1 (3.3)	2 (10)	1 (12.5)	0.55	1.0
Mortality, *n* (%)	1 (3.3)	2 (10)	2 (25)	0.55	0.55

*P*‐value 1—Group 1 *versus* Group 2, *P*‐value 2—Group 2 *versus* Group 3.

APACHE, Acute Physiology, Age, Chronic Health Evaluation; BISAP, Bedside Index of Severity in Acute Pancreatitis; CTSI, computed tomography severity index; CVS, cardio vascular system.

### *Clinical and severity scores*

BISAP scores were 2.13 ± 0.7, 2.3 ± 0.6, and 2.25 ± 0.7 in the conservative group, pigtail group, and surgery group, respectively, which did differ significantly. Modified computed tomography severity index (CTSI) was 7.64 ± 1.54, 7.88 ± 1.6, and 7.75 ± 1.67 in in the three groups, respectively, which were not significantly different. At admission, APACHE II scores were 10.4 ± 4.13, 9.05 ± 3.57, and 9.38 ± 1.30 in the conservative group, pigtail group, and surgery group, respectively, which also did not differ significantly. The APACHE II scores at first pigtail insertion were 9.7 ± 2.79 and 10.37 ± 2.97 in the pigtail group and surgery group, respectively, which were not significantly different. The APACHE II score at the time of surgery was 7.5 ± 2.0 in the surgery group (Table 2).

The length of hospital stay in days was 12 ± 7.2, 21.1 ± 11.7, and 70.1 ± 24.2 in the conservative group, pigtail group, and surgery group, respectively. There was a significant difference between both the conservative and pigtail groups and the pigtail and surgery groups (*P* = 0.0002), which was the duration of treatment for severity of the disease. Overall, 42 (72.4%) patients had pancreatic necrosis, with 23 (76.6%), 13 (65%), and 6 (75%) patients in the conservative, pigtail, and surgery groups, respectively, which did not differ significantly (Table 2).

### *Organ failure*

Renal failure was present in 16 (27.6%) patients overall, with 10 (33.3%) in the conservative group, 4 (20%) in the pigtail group, and 2 (25%) in the surgery group. Respiratory failure was the most common organ failure, with 40 (68.9%) patients in the overall study. It was distributed as follows: 19 (63.3%), 14 (70%), and 7 (87.5%) patients in the conservative, pigtail, and surgery groups respectively. All organ failure data were found to be insignificant within the groups (Table 2).

We also compared the mean of the total number of organs failed, which was 0.96 ± 0.76 in the conservative group, 0.9 ± 0.64 in the pigtail group, and 1.12 ± 0.83 in the surgery group; the median was one organ failure in each group, which did not differ significantly between the groups. Single organ failure was present in 30 (51.7%) patients overall, with 13 (43.3%) in the conservative group, 12 (60%) in the pigtail group, and 5 (62.5%) patients in the surgery group. A total of 13 (22.4%) patients had multiorgan failure (MOF), with 8 (26.7%), 3 (15%), and 2 (25%) patients in each group, respectively. The above three datasets were not significant statistically regarding a comparison between the three groups (Table 2). The extent of fluid collection and reduction after pigtail catheter drainage (PCD) was not compared in the intervention group.

### *Mortality*

A total of five deaths occurred during hospital stay (Table 2). Two (25%) patients in the surgery group died due to sepsis and organ failure. Two (10%) patients in the pigtail alone group died after pigtail insertion due to sepsis. Both the patients had MOF and were unfit for surgery. One (3.3%) patient died in the conservative group due to sepsis with acute renal failure on chronic kidney disease requiring multiple hemodialysis. The difference in mortality between the groups was insignificant.

### *Characteristics of pigtail catheter*

A total of 37 pigtail catheters were used in 28 patients, 26 in 20 patients of pigtail alone group, and 11 in 8 patients of surgery group. The mean number of pigtails per patient was 1.3 ± 0.57 and 1.37 ± 0.51 in the pigtail alone group and surgery group, respectively, which was not significantly different. Mean total duration of pigtail was 13.5 ± 5.7 and 24.1 ± 13.5 days in the pigtail alone and surgery groups, respectively, which were insignificant. The median duration was 12 and 24 days, respectively (Table 3). All PCD insertion was performed by surgeons, with the assistance of the duty radiologist.

**Table 3:** Characteristics of pigtail catheter and microbiology

Characteristics	Group 2 (*n* = 20)	Group 3 (*n* = 8)	*P*‐value
Total number of pigtails	26	11	—
Number of pigtails per patient (mean ± SD)	1.3 ± 0.57	1.3 ± 0.5	0.6
Duration of pigtails (mean ± SD)	13.5 ± 5.7	24.1 ± 13.5	0.06
Duration of pigtails—median (range)	12 (5–23)	24 (2–40)	—
Catheter‐related complications	0	3 (37.5)	0.01*
Infected pancreatic necrosis, *n* (%)	13 (65)	8 (100)	0.07
Unimicrobial, *n* (%)	11 (55)	8 (100)	0.02*
Polymicrobial, *n* (%)	2 (10)	0	1.0
Bacteria, *n* (%)			
*Escherichia coli*	7 (35)	5 (62.5)	0.23
*Klebsiella*	3 (15)	0	0.50
*Pseudomonas*	1 (5)	1 (12.5)	0.49
*Staphylococcus aureus*	1	1	—
*Enterobacter*	1	1	—
*Proteus*	1	0	—
*Enterococcus*	1	0	—

* Significant difference.

Catheter‐related complications were present in two patients with GI fistula and one patient of bleeding belonging to surgery group. All complications occurred preoperatively and were an indication for surgery. The complication rate was statistically significant between the two groups. No catheter‐related complications occurred in the pigtail alone group (Table 3).

### *Microbiology*

Infected pancreatic necrosis was present in 21 of 28 (75%) patients, with 13 (65%) in the pigtail alone groups and 8 (100%) patients in surgery group, which were not statistically significant. The infection was unimicrobial in 19 (67.8%) patients, with 11 (55%) patients in the pigtail alone group and 8 (100%) patients in the surgery group, which were significantly different. Polymicrobial infection was present in two patients, both belonging to the pigtail alone group. *Escherichia coli* was most common organism present in the culture. A total of 12 (42.8%) patients had grown *E. coli*, with 7 (35%) in the pigtail alone group and 5 (62.5%) in the surgery group, which was not significantly different. The second most common organism cultured was *Klebsiella*, with three patients all belonging to the pigtail alone group (Table 3).

### *End‐points*

The primary end‐point of sepsis reversal with pigtail insertion between the two groups was 78.5% (22 of 28 patients), with 18 (90%) in the pigtail alone group and four (50%) in the surgery group, which was statistically significant, with *P* = 0.03. Overall sepsis reversal within 1 week was 39.2% (11 of 28), with 50% (10 of 20) in the pigtail alone group and 12.5% (1 of 8) in the surgery group. Sepsis reversal with pigtail alone within 2 weeks was 64.2% (18 of 28), with 80% (16 of 20) in the pigtail alone group and 25% (2 of 8) in the surgery group, which was statistically significant between the two groups, with *P* = 0.01 (Table 4).

**Table 4:** End‐points

Characteristics	Group 2 (*n* = 20)	Group 3 (*n* = 8)	*P*‐value
Total number of pigtails	26	11	—
Sepsis reversal within 1 week of first pigtail alone, *n* (%)	10 (50)	1 (12.5)	0.09
Sepsis reversal within 2 weeks of first pigtail alone, *n* (%)	16 (75)	2 (25)	0.01*
Overall sepsis reversal with pigtail alone, *n* (%)	18 (90)	4 (50)	0.03*
Days of sepsis reversal after first pigtail (mean ± SD)	8.3 ± 3.2	20.3 ± 15	—
Days of sepsis reversal after first pigtail, median (range)	7 (4–15)	16 (7–49)	0.008*
Mortality, *n* (%)	2 (10)	2 (25)	0.55
Cather related complications	0	3 (37.5)	0.01*

* Significant difference.

A total of eight patients required surgery after pigtail insertion. Indications for surgery was catheter‐related complications in three patients (two GI fistula and 1intra‐abdominal bleeding), persisting/worsening sepsis and ongoing sepsis in three patients, incomplete drainage in one patient, and failure to thrive in one patient. Four patients (of 8) had sepsis reversal before the surgery, and pancreatic necrosectomy was performed in the same sitting.

### *Factors predicting surgery*

We conducted a univariate analysis of 16 factors using logistic regression for surgical intervention. Sepsis reversal within 2 weeks of pigtail insertion was statistically significant for predicting surgery, with *P* = 0.012. The other 15 factors studied were nonsignificant (Table 5).

**Table 5:** Factors predicting surgical intervention

Factors (univariate analysis)	Surgery odds ratio (95% CI)	*P*‐value
Age	0.978 (0.91–1.05)	0.54
Alcohol abuse	0.571 (0.06–5.19)	0.62
BISAP score	1.121 (0.36–3.45)	0.84
Modified CTSI	1.005 (0.61–1.63)	0.98
APACHE II initial	0.962 (0.77–1.19)	0.73
APACHE II at first pigtail	1.091 (0.83–1.46)	0.56
Number of organs failed	1.421 (0.51–3.95)	0.50
Multiorgan failure	1.974 (0.22–17.81)	0.55
Renal failure	1.167 (0.21–6.48)	1.16
Respiratory failure	0.277 (0.03–2.44)	0.24
Sepsis reversal within 1 week with pigtail alone	7.000 (0.72–67.84)	0.09
Sepsis reversal within 2 weeks with pigtail alone	12.000 (1.72–83.46)	0.012*
Total pigtails per patient	1.284 (0.29–5.61)	0.74
Pancreatic necrosis (>50%)	0.769 (0.13–4.40)	0.76
Total microorganisms per patient	2.513 (0.47–13.43)	0.28
*Escherichia coli*	0.323 (0.06–1.77)	0.19

* Significant difference.

APACHE, Acute Physiology, Age, Chronic Health Evaluation; BISAP, Bedside Index of Severity in Acute Pancreatitis; CI, confidence interval; CTSI, computed tomography severity index.

## Discussion

The management of infected acute pancreatitis had been an ever‐evolving field. Freeny *et al.* were the pioneers in this trend, who initially treated a series of patients with the only CT‐guided percutaneous catheter drainage.^5^ The other important studies with a sizeable number of patients were the PANTER trial,^6^ which compared a minimally invasive step‐up approach to conventional open necrosectomy in a randomized controlled trial by Dutch pancreatitis study group,^8^ and a prospective study by Babu *et al.,* 9 who managed a series of patients with severe acute pancreatitis by step‐up approach.

Freeny *et al.* 5 studied the outcome of a single alternative approach, which was percutaneous catheter drainage. The PANTER study^6^ was between two groups of patients managed by a minimally invasive step‐up approach and open necrosectomy. The Dutch pancreatitis group study,^8^ which did not give weight to a particular mode of intervention, and Babu *et al.* 9 actively managed patients of severe acute pancreatitis using the step‐up approach.

The primary end‐point of this study, which was overall sepsis reversal due to pigtail alone, was 78.5%, which was superior and closer to Freeny's study^5^ (74%) and Babu *et al.* 9 (62.5%). The PANTER^6^ and Dutch studies^8^ did not mention sepsis reversal with percutaneous drainage alone, although they mentioned the percentage of patients cured by percutaneous drainage alone, which was 35% in each. The therapeutic efficacy of pigtail drainage was found to be 64.2%, which was superior to other groups, such as Freeny^5^ (47%) and Babu *et al.* 9 (48.2%). Early presentation could be the explanation for a large number of patients in the conservatively managed group, which was 51.7%, unlike Babu *et al.* 9 (20%), where many were referred with delays of up to 10 days after the onset of disease. Conservatively managed patients were 62% in Dutch study,^8^ and no patient was from either Freeny's study^5^ or the PANTER study.^6^

We also noted a difference while comparing the incidence of persistent organ failure, which was 74% in our study, lower than Freeny's study,^5^ which reported 82.3% incidence organ failure, although they did not mention whether it was persistent or transient. Babu *et al.* 9 reported 51% incidence of persistent organ failure, and it was 33% in the Dutch study.^8^

The PANTER study^6^ did not mention organ failure before the intervention. When we looked at the incidence of MOF, it was again the highest in Freeny's study,^5^ with a value of 53%, while we observed a 22.4% incidence of MOF, which was comparable to the Dutch study^8^ (25%) and Babu's study^9^ (25.7%). The incidence of MOF in patients of surgery group was 25%, which was much lower compared to Freeny's study^5^ (55.5%) and Babu's study^9^ (56%), which contradicts the notion by Babu *et al.* that, in the case of MOF, more patients require surgical intervention. Respiratory failure was most common; 69% patients had respiratory failure in our study, which was comparable to Freeny's^5^ (67.6%) and was higher than Babu's^9^ (43%). Overall, we could not find any significant relation between organ failure or MOF and patients undergoing surgery.

Freeny^5^ reported that the overall mortality of 15 and 11.7% was disease specific, all of which occurred after surgery. The Dutch study^8^ reported 15% mortality overall, 17% in drainage alone, and 24% in the surgery group. The PANTER study^6^ reported 17% overall mortality, 12% in drainage alone, and 16% in the surgery group. Babu *et al.* 9 reported overall mortality of 24.2% and disease‐specific mortality of 20%. They also reported 6.8% mortality in drainage alone and 40.7% in the surgery group. The low overall mortality in our study can be explained by the fact that our patients presented early to the hospital as opposed to Babu's study,^9^ and a large proportion of them was cured by conservative management alone. However, the exact median time of presentation has not been studied. Mortality in the pigtail group was comparable to the Dutch study,^8^ and mortality in surgery group was comparable to the PANTER study.^6^

When we compared the incidence of catheter‐related complications, we noted that Freeny *et al.* 5 reported none, and it was 10.7% in our study (3/28), which was similar to Babu *et al.,* 9 10.7% (6/56). The complications were colonic fistula in two patients (7.1%) managed by diverting ileostomy and intra‐abdominal bleeding in a patient (3.6%) who required laparotomy. Colonic fistula patients had a reversal of sepsis before surgery, while the bleeding occurred after 2 days of drain insertion. All three patients underwent necrosectomy in the same setting.

In our study, the incidence of infected pancreatic necrosis was 75% compared to 94% in Freeny's study,^5^ 31.6% in the Dutch study,^8^ and 75.7% in Babu's study.^9^ It could have been higher if we performed routine aspiration of acute pancreatic fluid collection, unless the patient was planned for pigtail insertion. However, it was comparable to Babu's study.^9^ The incidence of infection was polymicrobial in 10% of infected cases as opposed to 60% in Babu *et al.* 9

Another important finding was that 100% of patients in the surgery group had a unimicrobial infection. In our study, *E. coli* was the cause of infection in 57% cases of infected pancreatic necrosis, while Babu *et al.* 9 reported an association of 71.6%. High association of *E. coli* with infected pancreatic necrosis has been reported in other studies as well.^10^, ^11^, ^12^, ^13^, ^14^ The reason for this is the infection by translocation of the bacteria from the gut.^15^

Overall, 64.2% patients achieved sepsis reversal within 2 weeks, which was a significant finding. While testing the factors predicting surgery using logistic regression in the univariate analysis, we found that sepsis reversal within 2 weeks had a significant correlation. Babu *et al.* 9 had considered this cut‐off duration to be 1 week, which was found to be nonsignificant in our study because a sizeable number of patients not only had sepsis reversal between the first and second week of pigtail insertion, but they were also cured completely. On univariate analysis, we analyzed several other factors, but none were found to be significant.

### *Limitation*

This study had a smaller sample size and was a single‐center study.

In conclusion, we achieved significant sepsis reversal by pigtail catheter drainage, and most of the patients recovered completely without requiring any surgical intervention. The step‐up approach avoided unnecessary intervention, and half of the patients recovered by conservative treatment alone. Absence of sepsis reversal within 2 weeks of pigtail insertion can be used as a predictor of surgery in the early course of severe acute pancreatitis managed using the step‐up approach.
